# Novel *ITGB6* variants cause hypoplastic-hypomineralized amelogenesis imperfecta and taurodontism: characterization of tooth phenotype and review of literature

**DOI:** 10.1038/s41405-023-00142-y

**Published:** 2023-04-11

**Authors:** Kanokwan Sriwattanapong, Thanakorn Theerapanon, Lawan Boonprakong, Anucharte Srijunbarl, Thantrira Porntaveetus, Vorasuk Shotelersuk

**Affiliations:** 1 Center of Excellence in Genomics and Precision Dentistry, Department of Physiology, Faculty of Dentistry, Chulalongkorn University, Bangkok, 10330 Thailand; 2 Office of Research Affairs, Faculty of Dentistry, Chulalongkorn University, Bangkok, 10330 Thailand; 3 Dental Materials R&D Center, Faculty of Dentistry, Chulalongkorn University, Bangkok, 10330 Thailand; 4 Center of Excellence for Medical Genomics, Department of Pediatrics, Faculty of Medicine, Chulalongkorn University, Bangkok, 10330 Thailand; 5 Excellence Center for Genomics and Precision Medicine, King Chulalongkorn Memorial Hospital, the Thai Red Cross Society, Bangkok, 10330 Thailand

**Keywords:** Oral diseases, Dentistry

## Abstract

**Objectives:**

To characterize phenotype and genotype of amelogenesis imperfecta (AI) in a Thai patient, and review of literature.

**Materials and methods:**

Variants were identified using trio-exome and Sanger sequencing. The ITGB6 protein level in patient’s gingival cells was measured. The patient’s deciduous first molar was investigated for surface roughness, mineral density, microhardness, mineral composition, and ultrastructure.

**Results:**

The patient exhibited hypoplastic-hypomineralized AI, taurodontism, and periodontal inflammation. Exome sequencing identified the novel compound heterozygous *ITGB6* mutation, a nonsense c.625 G > T, p.(Gly209*) inherited from mother and a splicing c.1661-3 C > G from father, indicating AI type IH. The ITGB6 level in patient cells was significantly reduced, compared with controls. Analyses of a patient’s tooth showed a significant increase in roughness while mineral density of enamel and microhardness of enamel and dentin were significantly reduced. In dentin, carbon was significantly decreased while calcium, phosphorus, and oxygen levels were significantly increased. Severely collapsed enamel rods and a gap in dentinoenamel junction were observed. Of six affected families and eight *ITGB6* variants that have been reported, our patient was the only one with taurodontism.

**Conclusion:**

We report the hypoplasia/hypomineralization/taurodontism AI patient with disturbed tooth characteristics associated with the novel *ITGB6* variants and reduced ITGB6 expression, expanding genotype, phenotype, and understanding of autosomal recessive AI.

## Introduction

Dental enamel is an epithelial-derived tissue comprising of organized hydroxyapatite crystals configured into enamel rods [1]. Amelogenesis in mouse has been described in four defined stages: presecretory, secretory, transition, to maturation stages [2]. In the presecretory stage, the ameloblasts acquire their characteristics and begin secreting enamel matrix proteins. In the secretory stage, the entire thickness of the enamel layer is established. In the transition and maturation stages, the ameloblasts restructure and transport mineral ions into the enamel fluid, promoting the inorganic content and growth of enamel prisms [3]. An entire process of amelogenesis is under genetic control. Alterations in genes responsible for amelogenesis at different timings result in enamel malformations with diverse phenotypes [4].

Amelogenesis imperfecta (AI) is a heterogeneous group of genetic conditions characterized by defects in enamel formation and affecting both deciduous and permanent teeth. The Witkop’s classification categorized AI into four main types: (1) hypoplastic, (2) hypomaturation, (3) hypocalcification, and (4) hypomaturation-hypoplastic with taurodontism, and subdivided into fifteen subtypes (IA-IG, IIA-IID, IIIA-IIIB, and IVA-IVB) by phenotype and inheritance pattern [5]. The Online Mendelian Inheritance in Man (OMIM) has associated specific genes with phenotypes and expanded the AI designations into: AI type IA (*LAMB3*), IB and IC (*ENAM*), IE (*AMELX*), IF (*AMBN*), IG (*FAM20A*), IH (*ITGB6*), IJ (*ACP4*), IK (*SP6*), IIA1 (*KLK4*), IIA2 (*MMP20*), IIA3 (*WDR72*), IIA4 (*ODAPH*), IIA5 (*SLC24A4*), IIA6 (*GPR68*), III1 (*FAM83H*), IIIB (*AMTN*), IIIC (*RELT*), and IV (*DLX3*) [6].

The integrin beta-6 *(ITGB6)* gene (OMIM *147558) comprises fifteen exons and encodes the β6 subunit of the integrin αvβ6 that plays an important role in cell-cell and cell-matrix adhesion, cellular proliferation and migration, tissue repair, apoptosis, inflammation, and angiogenesis [7, 8]. It also activates extracellular matrix protein and transcription factors such as fibronectin, tenascin-C, vitronectin, latency-associated peptide of transforming growth factor- β1 (TGF- β1), and TGF- β3 [9, 10]. The integrin αvβ6 is expressed in the ameloblasts and regulates enamel biomineralization, amelogenin deposition, and TGF-β1 that controls cell growth and differentiation during early tooth development [11, 12]. The expression of *Itgb6* is detected in the differentiating ameloblasts, secretory ameloblasts, and maturation ameloblasts, indicating its involvement in amelogenesis [13]. In the *Itgb6*^−/−^ mice, an accumulation of amelogenin in enamel extracellular matrix was disturbed, resulting in a reduction in enamel mineralization [14], that mimics the human AI phenotype. The biallelic mutations in the *ITGB6* gene lead to hypoplastic and/or hypomineralized AI (AI type IH) (MIM #616221) [9, 13, 15].

Currently, the knowledge of genotypes and phenotypes related to the *ITGB6* are still elusive. There have been only five families and six different *ITGB6* mutations reported. Only one study demonstrated the scanning electron microscopic images of one affected tooth [9]. Identifying more patients and new *ITGB6* variants helps understand the biological role of *ITGB6* in humans and establish phenotype-genotype correlation benefiting disease management.

In this study, we identified a Thai family with the proband having AI, taurodontism, and periodontal inflammation. Trio-exome and Sanger sequencing was performed to detect pathogenic variants. The patient’s tooth was investigated for its physical and mechanical properties and ultrastructure. The patient’s gingival fibroblasts were investigated for the ITGB6 protein level. A review of literature of all reported *ITGB6* cases and mutations were performed.

## Materials and methods

### Subjects

This study was approved by the Institutional Review Board, Faculty of Medicine, Chulalongkorn University (IRB 813/63) and in accordance with the 1964 Helsinki declaration and its later amendments. A written informed consent for participation in the study and publication of information was obtained from each participant or a legal guardian. Medical examination and laboratory investigation were performed.

### Genetic variant analysis

Genomic DNA was extracted from peripheral blood leukocytes of patients using Puregene Blood Kit (Qiagen, Hilden, Germany) and sent for whole exome sequencing using illumina Hiseq 2000 Sequencer (Macrogen, Seoul, Korea). The sequences were aligned using University of California Santa Cruz (UCSC) hg19 and Burrows–Wheeler Aligner (http://bio-bwa.sourceforge.net/). Downstream process was performed by SAMtools (samtools.sourceforge.net/) and annotated against dbSNP and 1000 Genomes. The variants were filtered out using the following criteria: (1) coverage < 10x; (2) minor allele frequency >1% in the 1000 Genomes Project, and Genome Aggregation Database (gnomAD: gnomad.broadinstitute.org); (3) present in the Thai reference exome (T-REx) variant database [16], (4) synonymous exonic variants; and (5) not located in the coding region and splice site of genes related to AI (HP:0000705) (Table S1) [17]. The filtered variants were called novel if they were not detected by searching in Google scholar (https://scholar.google.com/), Varsome [18] and Mastermind Genomic Search Engine (https://www.genomenon.com/mastermind).

The identified *ITGB6* variants were confirmed by Sanger sequencing using the primers (NM_000888.3) c.625 G > T: F′TAGGAGAATGTTGCTAAGCT and R′AGAGTGAAACAGCACTCCCT and c.1661-3 C > G: F’TGGAGAAAAGCAGAGACATTACC and R′CTCATACTGCACCCCTCACAC.

### In silico analysis and pathogenicity prediction

Mutation taster, Human splicing finder (http://www.umd.be/HSF3/credits.html), Combined Annotation Dependent Depletion (CADD) v.16 software (https://cadd.gs.washington.edu/), and varSeak (www.varseak.bio) were used to predict the effects of the *ITGB6* variants [19–21]. The pathogenicity of variants was classified according to the American College of Medical Genetics and Genomics (ACMG) standard guidelines [22].

### Cell isolation and culture

Fibroblasts were isolated from gingival tissues of the proband and three sex-matched Thai healthy individuals of the same age range who did not have any systemic diseases and orodental abnormalities (controls). Briefly, the gingival tissues were cut into 1 × 1 mm pieces and placed in a 35-mm culture dish (Corning, New York, USA). Cells were maintained in growth medium composed of Dulbecco’s Modified Eagle Medium (DMEM), 10% fetal bovine serum (Gibco, CA, USA), 1% L-glutamine (Gibco), and 1% penicillin and streptomycin (Gibco), and incubated in a humidified environment at 37 °C and 5% CO_2_. Cells from passages 5 were used in the experiments.

### Western blot analysis

Confluent monolayer cells were harvested, washed with ice-cold phosphate-buffered saline (PBS), and lysed in radioimmunoprecipitation buffer (RIPA) (Thermo Fisher Scientific, MA, USA) containing the Halt^TM^ protease inhibitor cocktail (Thermo Fisher Scientific, MA, USA). Protein concentration was determined using Pierce^TM^ BCA Protein Assay Kit (Thermo Fisher Scientific, MA, USA). A total protein (30 µg) was separated by 7.5% sodium dodecyl sulfate-polyacrylamide gel electrophoresis and transferred to a polyvinylidene difluoride membranes (PVDF) (Bio-Rad, CA, USA). The PVDF membranes were blocked with 5% BSA (Merck Millipore, Germany) in TBST buffer (10 mM Tris-HCl pH 8.0, 150 mM NaCl, and 0.1%Tween-20) for 1 h at room temperature, and probed with a primary antibody overnight at 4 °C. Mouse anti-human Integrin β6 monoclonal antibody (1:1000 dilution; Merck, Darmstadt, Germany, Cat No. MAB2076Z) and mouse anti-human GAPDH (1:3000 dilution; Abcam, Cambridge, UK, Cat No. ab8245) were used as the primary antibodies. The membranes were incubated with the HRP-conjugated secondary antibody; anti-mouse, (1:2500 dilution; R&D systems, MN, USA, Cat. No. HAF018) for 2 h at room temperature. They were treated with SuperSignal^TM^ West Femto Maximum Sensitivity Substrate (Thermo Fisher Scientific, MA, USA) and analyzed using the Amersham^TM^ Imager 680 (GE Healthcare, Illinois, USA). The intensity of the bands was quantified using ImageJ software.

### Tooth samples

A deciduous maxillary first molar of the proband was extracted according to dental treatment plan. The cervical enamel was remained after stainless steel crown was removed and used for further analyses, compared with three tooth type-matched molars obtained from age-matched healthy individuals.

### Micro-computerized tomography (micro-CT)

Teeth were scanned with Specimen Micro-CT35 (SCANCO Medical AG). Three spots (30 layers/ each spot) of enamel and dentin in the same location between the patient’s tooth and controls were selected to quantify mineral density. The images were processed using Image Processing Language (Scanco Medical AG).

### Surface roughness, microhardness, ultrastructure, and mineral composition

The same locations of the patient’s tooth and controls were selected. Surface roughness was measured for thirty spots every 600 µm using the Talyscan 150 and TalyMap Universal program (Taylor Hobson Ltd). Five spots of enamel and dentin were tested for microhardness using a microhardness tester (FM-700e Type D, FUTURE-TECH, Kanagawa, Japan). For ultrastructural analysis, tooth sections were etched with 37% phosphoric acids, rinsed, dried using a critical point dryer (Emitech K850), covered with gold powder (Jeol JFC-1200), and examined using the scanning electron microscope (SEM, Quanta FEG-250). The amount of the patient’s remaining enamel was not sufficient for Energy Dispersive X-ray (EDX) measurement. In dentin, the amount (%) of carbon (C), oxygen (O), phosphorus (P), and calcium (Ca) was measured for five locations using EDX (ISIS 300).

### Statistical analysis

Statistical analyses for tooth characterization and protein expression were performed using Mann–Whitney U test (*P* < 0.05) in a GraphPad Prism8 Software Inc.

## Results

### Patient phenotype

**Fig. 1:**
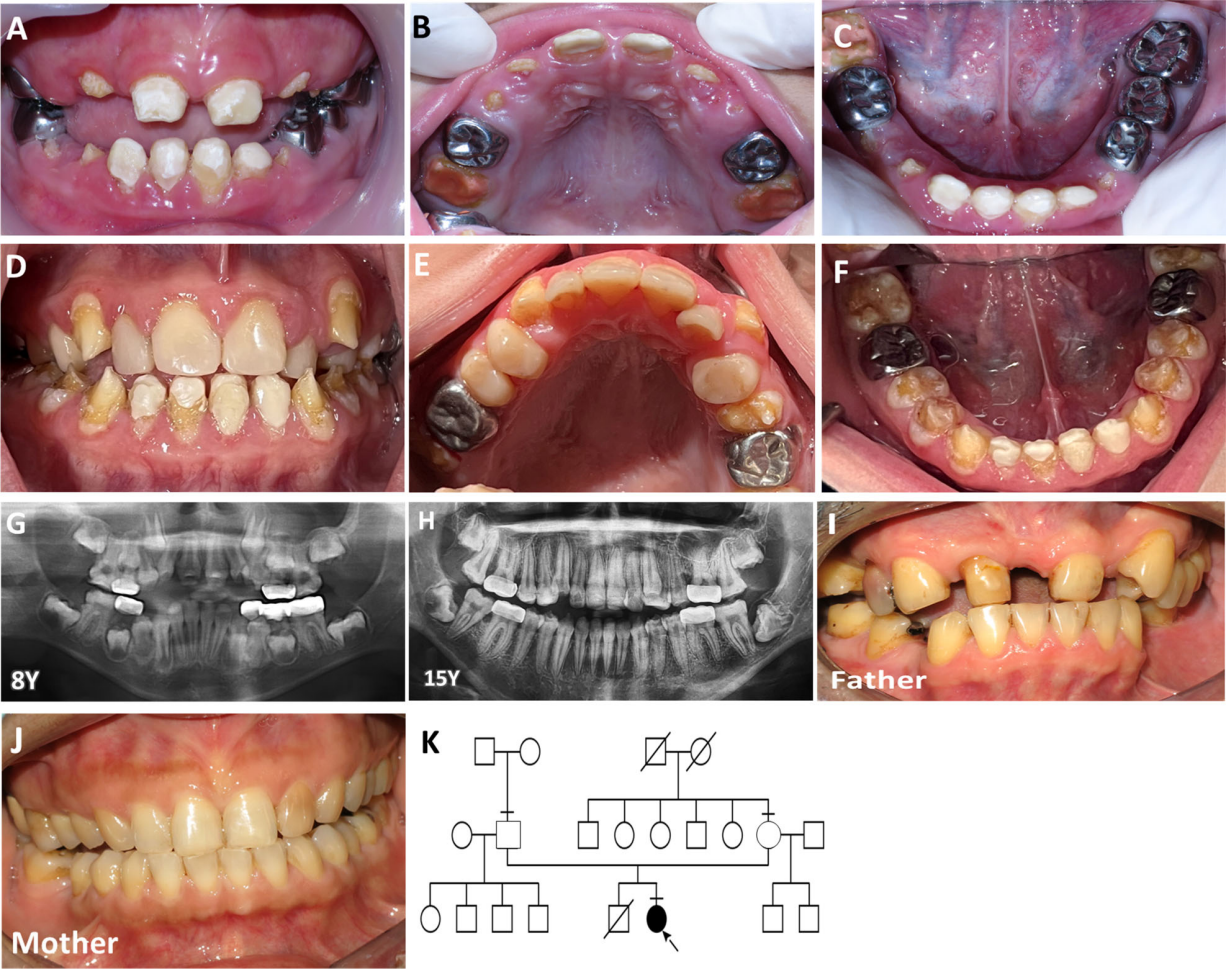
Clinical and radiographic feature, family pedigree, and chromatograms of the patient and family. **A**–**C** Orodental features of the proband at 8-year-old showed yellow teeth with rough and pitted enamel. The deciduous molars were restored with stainless steel crowns. **D**–**F** Orodental features of the proband at 15-year-old showed dark yellow teeth with rough enamel that was severely eroded. A band of enamel was observed along the cervical margin and cusp tips of the teeth. **G**, **H** Panoramic radiographs showed a reduction in thickness and radiodensity of enamel. Taurodontism was observed in the permanent maxillary second molars. The permanent first molars showed bone loss. **I**, **J** The parents did not have enamel defects. **K** Family pedigree showed that the proband (arrow) was the only person in the family with enamel malformation.

A proband, 6-year-old girl, presented with discolored teeth and anterior open bite. The patient had a history of frequent common cold since 3 months old and was admitted to the hospital due to pneumonia at the age of 13 years. Oral examination at age 8 years of age showed that the enamel on the erupting upper lateral incisors and lower canines were rough, pitted, and yellowish. The permanent incisors were restored using composite resin. The deciduous molars and permanent lower left first molar were restored with stainless steel crowns. The permanent upper and lower right first molars were covered with glass ionomer cement (GC Fuji VII) (Fig. 1A–C). At 15 years of age, the patient presented with severely eroded teeth with the remaining enamel along the cervical margin and cusp tips of teeth. The incisors and upper premolars were restored with composite resin and the first molars with stainless steel crowns. Generalized gingival inflammation was clinically observed (Fig. 1D–F). Panoramic radiographs showed a reduction in thickness and radiopacity of enamel. Hypertaurodontism was observed in the permanent maxillary second molars. Alveolar bone loss associated with the first molars was evidence. Dental development was within normal limit (Fig. 1G, H). The parents were healthy and not consanguineous. The father had a history of tooth extraction. Other family members did not have AI (Fig. 1I–K).

### Genetic variants and protein expression

**Fig. 2:**
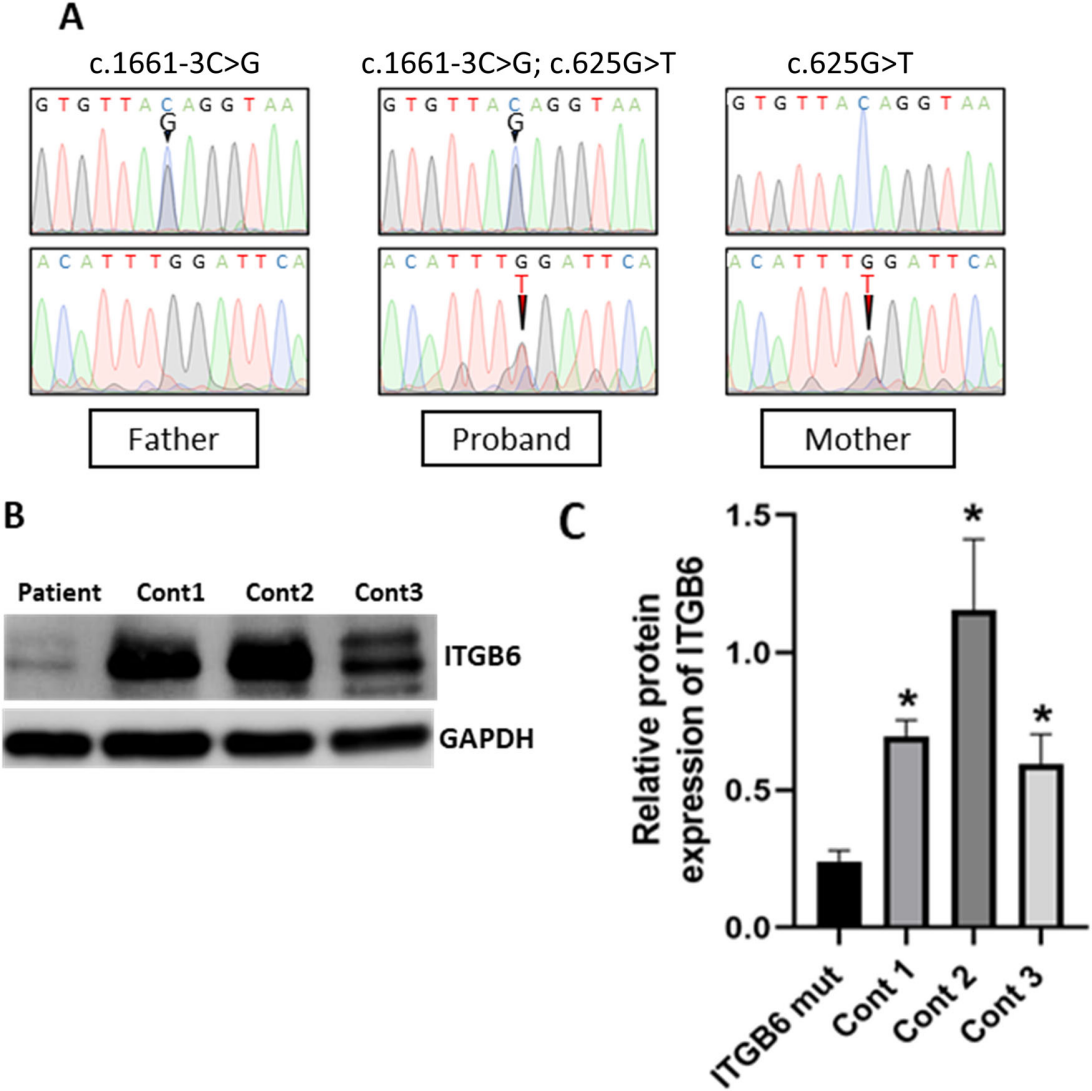
Genetic analysis and protein expression. **A** Chromatogram demonstrated the compound heterozygous variants in the proband. The c.625 G > T, p.(Gly209*) was inherited from the mother and the c.1661-3 C > G from the father. **B**, **C** The ITGB6 level in the patient’s gingival cells was significantly lower than that in controls.

Trio-exome sequencing identified that the proband harbored the compound heterozygous variants in *ITGB6*: the nonsense variant c.625 G > T, p.(Gly209*) (ClinVar: SCV002058096) in the exon 5 was inherited from mother and the splicing variant c.1661-3 C > G (SCV002058097) in the intron 11 was inherited from father (Fig. 2A). No other variants in genes related to AI (HP:0000705) passed the filtering criteria. These indicate AI type IH (MIM #616221). Both identified variants have not been associated with any clinical phenotype of defective enamel. In silico analysis using Mutation taster and Human Splicing Finder predicted that the ENST00000409872: c.1661-3 C > G resulted in the broken acceptor splice site of the intron 11 of *ITGB6* that might affect splicing (Table S2). The varSEAK predicted that the c.1661-3 C > G variant could affect mRNA splicing and cause exon skipping. The CADD scores of the c.625 G > T and c.1661-3 C > G variants were 39 and 24.4, and, according to ACMG guideline, classified as pathogenic and likely pathogenic, respectively. Western blot results showed that the level of ITGB6 protein in the patient’s gingival cells was significantly lower than that in controls (Fig. 2B, C).

### Properties and ultrastructure of the patient tooth

**Fig. 3:**
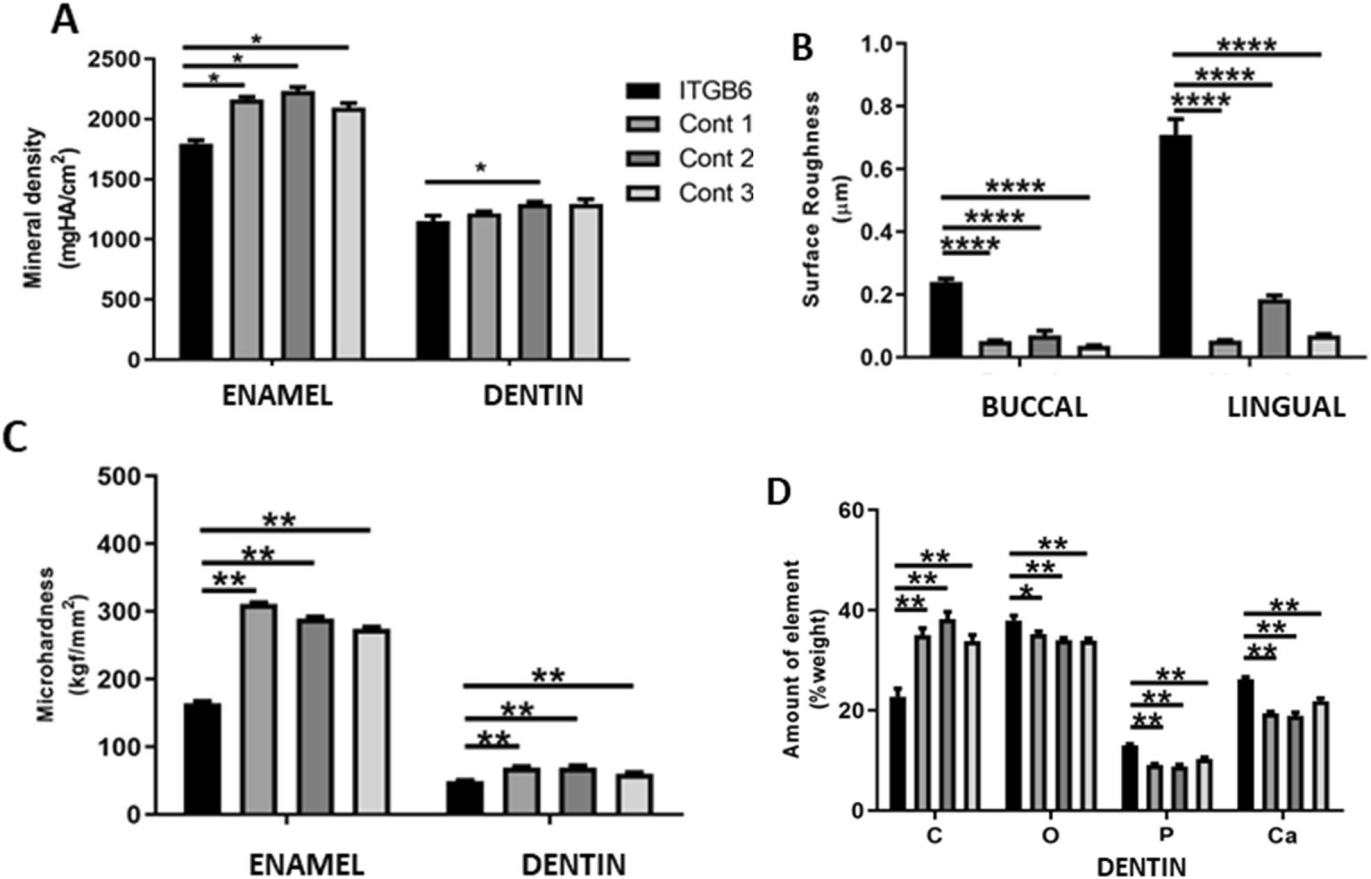
Tooth characteristics. **A** The patient’s tooth showed a significant reduction in mineral density compared with controls. **B** Surface roughness values of the patient’s tooth was significantly higher than those of controls. **C** Microhardness values of the patient’s enamel and dentin were significantly reduced compared with those of controls. **D** The patient’s dentin demonstrated a significantly decrease in carbon content, but significant increases in oxygen, phosphorus, and calcium levels, compared with those in controls.

Micro-CT analysis showed that mineral density of the patient’s enamel was significantly reduced while its dentin mineral density was comparable to those of controls (Fig. 3A). The surface roughness of the patient’s tooth was significantly increased, compared with those of controls (Fig. 3B). The microhardness of the patient’s enamel and dentin was significantly reduced, compared with those of controls (Fig. 3C). The patient’s dentin showed a significant decrease in carbon, but significantly increases in oxygen, phosphate, and calcium levels, compared with those in controls (Fig. 3D).

**Fig. 4:**
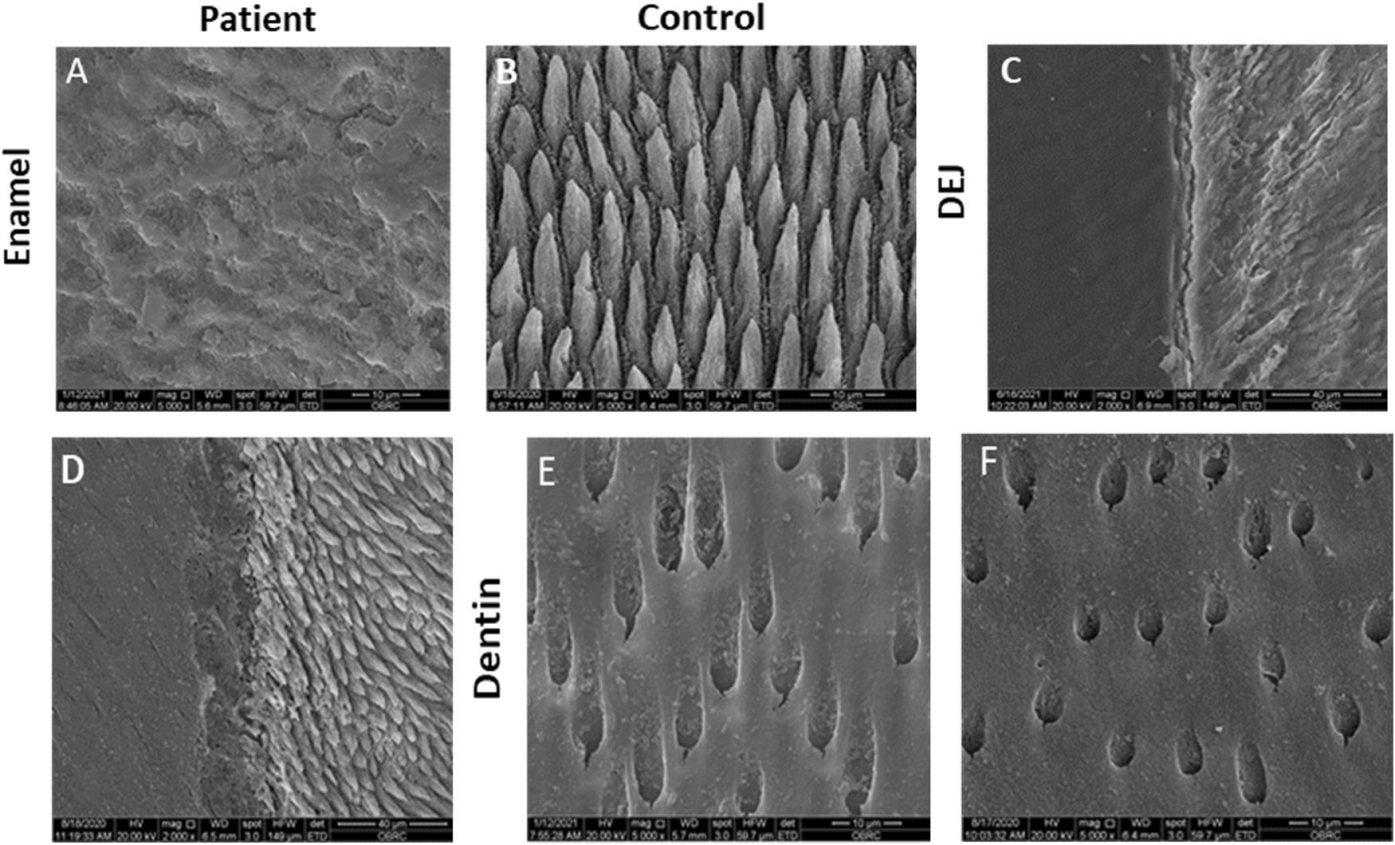
Ultrastructure of *ITGB6* tooth. **A**, **B** SEM showed disorganized, flatted, and collapsed enamel rods in the patient’s tooth while an organized arrangement of enamel rods was observed in control. **C**, **D** A gap in the dentinoenamel junction of *ITGB6* tooth was observed while control showed a smooth connection between enamel and dentin. **E**, **F** The dentinal *ITGB6* was similar to that of the control teeth.

SEM demonstrated that the patient’s enamel rods were severely disorganized, coarse, and collapsed while controls showed typically organized enamel rods (Fig. 4A, B). The patient’s tooth displayed a gap in the dentinoenamel junction (DEJ) while the control exhibited a smooth and continuous connection between enamel and dentin (Fig. 4C, D). Regarding the ultrastructure, the patient’s dentin was comparable to the controls (Fig. 4E, F).

## Discussion

Using trio-exome sequencing, the patient was found to be a compound heterozygote for the nonsense c.625 G > T p.(Gly209*) and the splicing c.1661-3 C > G in the *ITGB6* gene while the parents were heterozygous for each variant, indicating autosomal recessive AI. The identified genetic mutations together with the feature of hypoplastic and hypomineralized AI designates AI type IH.

**Fig. 5:**
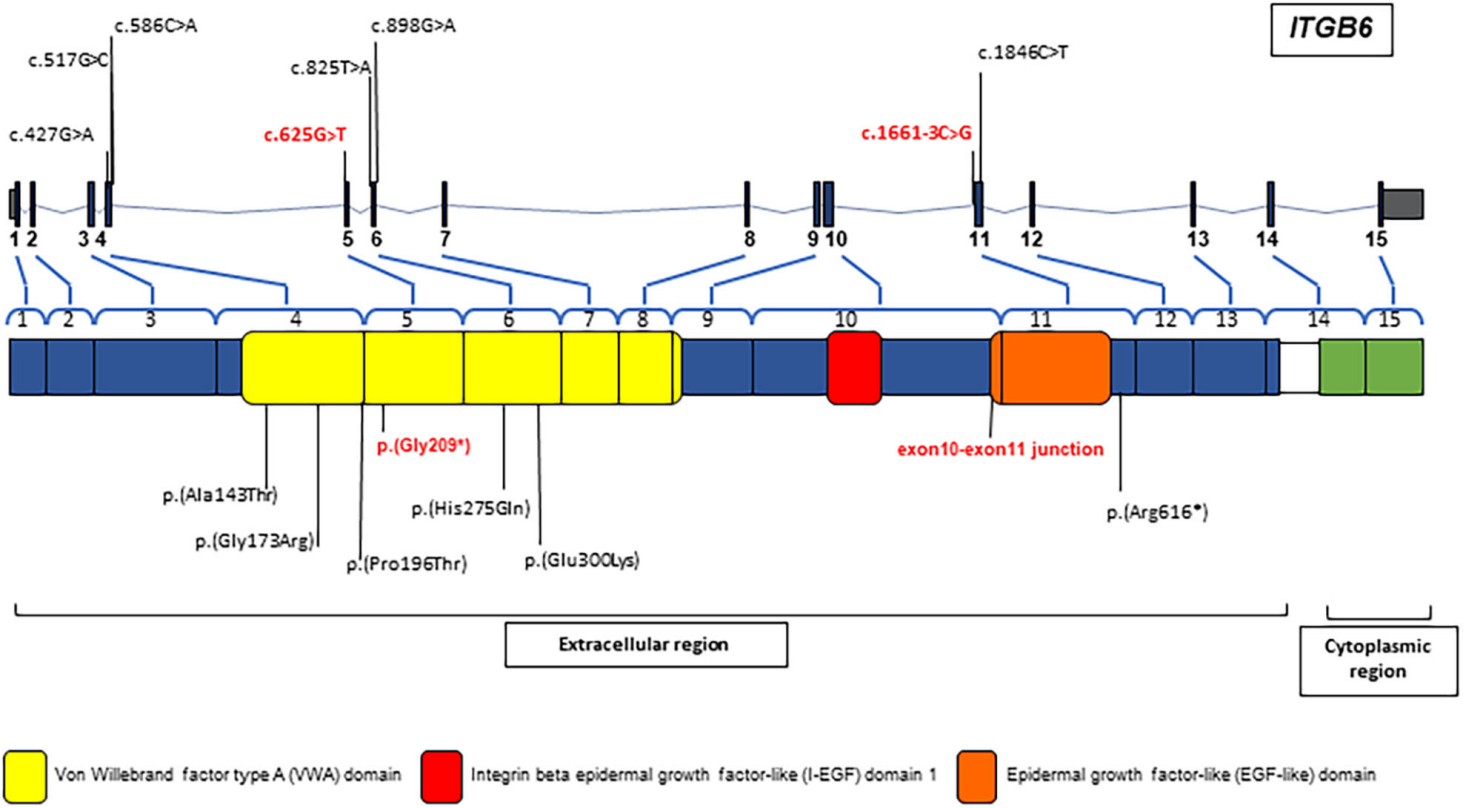
Schematic diagram of the *ITGB6* gene and protein. In both schemes, all *ITGB6* variants identified to date, which associated with enamel malformation, are shown. The variants detected in this study are in bold texts.

To the best of our knowledge, six affected families and eight *ITGB6* mutations (including a family in this study) have been identified [9, 13, 15, 23]. Table 1 and Fig. 5 summarized the genotype and phenotype of all *ITGB6* affected individuals. Our patient was the only one who had taurodontism. All *ITGB6* affected individuals shared common features of hypoplastic and/or hypomineralized AI. The teeth were rough, pitted, and yellowish. These except the patients reported by Ansar et al., 2016 who showed only rough enamel surface, not a typical AI feature [23]. A Hutchinson/screwdriver tooth morphology was observed in a patient who was homozygous for the c.1846C > T, p.(Arg616*) and hemizygous for the c.1697T > C p.(Met566Thr) in *Nance-Horan syndrome* (*NHS*) [13].

**Table 1:** The patients identified with enamel defects and *ITGB6* variants.

Patient	Age (Y)	Nation/ Ethnic	Consanguinity	Inheritance	Nucleotide change	Amino acid change	Exon	Clinical features	Radiographic features
This study									
Proband	8	Thai	No	Cpd Het	c.625 G > T c.1661-3 C > G	p.(Gly209*)-	5 Intron11	- Pitted, rough, chalky and yellowish enamel -Hypomineralized and hypoplastic enamel -Generalized gingival inflammation -Anterior open bite	-Reduced radiopacity and thickness of enamel -Taurodontism: maxillary upper second molars
Wang et al., 2014									
Proband	8	Hispanic	No	Cpd Het	c.427 G > A c.825 T > A	p.(Ala143Thr) p.(His275Gln)	4	**-**Very thin/hypoplastic enamel, rough surface **-**Anterior open bite, Class III malocclusion, attrition	Thin and normal contrasting layer of enamel (unerupted teeth)
(Family1)							6		
Proband	8	Hispanic	No	Homo	c.1846C > T	p.(Arg616*)	11	**-**Yellow-brown, rough, hypoplastic enamel **-**Thin enamel collar at the cervical margin **-**Attrition **-**Hutchinson/screwdriver maxillary incisors **-**A hemizygous c.1697T > C p.(Met566Thr) in the NHS Actin Remodeling Regulator or Nance-Horan Syndrome (*NHS*).	-Thin and contrasting layer of enamel (Unerupted teeth) -Missing mandibular left second molar
(Family2)									
Poulter et al., 2013									
V:3	7	Pakistan	Yes	Homo	c.586 C > A	p.(Pro196Thr)	4	**-**Pitted, rough, hypomineralized enamel with pigmentation **-**Remaining enamel on the molar cusp tips and cervical margin **-**Reduced mineral density and voids in enamel **-**Disturbed orientation of enamel prisms	Near-normal volume and contrasting enamel (unerupted teeth)
Seymen et al., 2015									
Proband	8	Turkish	Yes	Homo	c.517 G > C	p.(Gly173Arg)	4	**-**Thin, pitted, pigmented, hypoplastic and hypomineralized enamel. **-**Thicker enamel in the cervical area of molars	Reduced thickness and radiopacity of enamel
Ansar et al., 2016									
IV-2	33	Pakistan	Yes	Homo	c.898 G > A	p.(Glu300Lys)	6	**-**Adolescent alopecia, intellectual disability **-**Rough enamel, yellowish-brown staining, malocclusion, gingival recession, tooth loss **-***AI feature was not definite*.	Not available
IV-3	39								
IV-6	40								

*Cpd Het* compound heterozygous, *Homo* homozygous, *Hemi* hemizygous, *Y* Year.

Four families were found to be homozygous, and two families were compound heterozygous for the variants. Eight identified *ITGB6* mutations consist of 5 missense, 2 nonsense, and 1 splicing. Of the six previously reported variants: three were in the exon 4, two in the exon 6, and one in the exon 11. The p.(Gly209*) detected in our patient was the first variant identified in the exon 5 and the c.1661-3 C > G was the first splicing variant near the exon 11 of *ITGB6*.

The ITGB6 protein contains the extracellular, transmembrane, and cytoplasmic regions. The extracellular region consists of the VWA domain, integrin beta epidermal growth factor-like (I-EGF) domain 1, and epidermal growth factor-like (EGF-like) domain. The nonsense p.(Gly209*) identified in our patient is located in the Von Willebrand factor A (VWA) domain that is important for interacting with the α subunit of ITGB. It is expected to be degraded by nonsense-mediated mRNA decay (NMD). Six out of eight identified variants are detected in the VWA domain, confirming that this domain is vital for the ITGB6 function. The splicing variant c.1661-3 C > G in the intron 11 is predicted to affect acceptor splice site and mRNA splicing, and might cause exon skipping. The expression of ITGB6 in the patient’s gingival cells were significantly reduced, compared with that in controls, suggesting that the mutations were loss-of-function. This finding is consistent with the *Itgb6*^*−/−*^ mice that were negative for the integrin beta 6 expression [14].

The *Itgb6*^*−/−*^ mice showed chalky teeth with reduced mineralization and severe attrition, mimicking hypomineralized AI [14]. Moreover, the *Itgb6*^*−/−*^ mice developed periodontal disease exhibiting epithelial inflammation, pocket formation, and alveolar bone loss [12]. The integrin αvβ6 is expressed in ameloblasts and junctional epithelium and plays a crucial role in regulating amelogenin deposition in enamel mineralization as well as protecting periodontal tissue against inflammation through activation of TGF-β1 [12, 14]. The EGF-like domain of ITGB6 contains the RGD integrin-binding motif that interacts with extracellular matrix proteins and TGF-β1. The TGF-β1 has been shown to involve not only in enamel mineralization but also periodontal inflammation [24, 25]. The periodontal inflammation observed in our patient might be a consequence of altered *ITGB6* functions or local factors such as stainless steel crowns and poor oral hygiene, or both genetic and local involvements. Identification of more *ITGB6* patients and further functional studies should be performed to validate the relationship between *ITGB6* and periodontal disease.

Additionally, *ITGB6* is highly expressed in the lungs and the *Itgb6* knockout mice exhibited significant airway inflammation, age-related emphysema, and juvenile baldness, that are related to *TGF-β1* deficiency [26, 27]. Unlike knockout mice, there have not been no reports of *ITGB6* patients with pulmonary inflammation. However, it is unclear whether a detailed medical examination of the reported patients was performed. AI may present with other medical problems such as skeletal anomalies, heart defects, nephrocalcinosis, and cone rod dystrophy [28–30]. Ansar et al., 2016 reported patients with *ITGB6* mutation who presented rough enamel, periodontal disease, intellectual disability, early-onset skin aging, and adolescent alopecia. Our patient also had periodontal inflammation, frequent common cold, and a history of pneumonia. These might suggest the possibility that *ITGB6* mutations might be the causes of other phenotypes of human disease not limited to AI.

Anterior open bite was observed in our patient and in the one with *ITGB6* variants reported by Wang et al., 2014 [13], indicating that open bite can be found in patients with *ITGB6* mutations. Clinical studies have shown that open bite is more commonly found in patients with AI than in the general population [31], particularly those who carry mutations in the *ENAM* or *AMELX* gene [32–34]. The relationship between AI and open bite remains unknown; however, it has been suggested that the etiology of open bite in individuals with AI may be due to a genetic factor that determines craniofacial development and alveolar growth [31, 33]. Taurodontism is classified into hypotaurodontism, mesotaurodontism, and hypertaurodontism [35]. It can occur as an isolated trait or as a part of genetic syndromes such as amelogenesis imperfecta (*AMELX* or *DLX3*), Down syndrome (Trisomy 21), ectodermal dysplasia (EDA) and osteogenesis imperfecta (*COL1A1* and *COL1A2*) [36–38]. Hypertaurodontism, which was observed in our patient, expands the phenotypic spectrum of *ITGB6*-related disorder.

Detailed analyses of the patient’s tooth showed a significant increase in surface roughness and significant decreases in enamel mineral density and microhardness of enamel and dentin. The enamel findings are consistent with the clinical feature of hypoplastic-hypomineralized AI and the hypomineralized enamel in *Itgb6*^*−/−*^ mice [14]. Unlike the study by Poulter et al., 2014 that showed the arrays of enamel prisms in the *ITGB6* tooth sample, the enamel rods of our patient’s tooth were severely collapsed. This is explained by the expression of *ITGB6* in the ameloblasts at the onset of enamel formation and similar to the *Itgb6* null mice that failed to form enamel rods [13]. In the patient’s dentin, the carbon level was significantly reduced while the oxygen, phosphate, and calcium levels were significantly increased, compared with those in controls. Moreover, the patient’s tooth exhibited a gap along the DEJ, suggesting the discontinuation between defective enamel and dentin. During tooth development, *ITGB6* is involved not only in enamel formation, but also in cell adhesion and cell-matrix interactions that are important in epithelial-mesenchymal interactions [13]. It is possible that the *ITGB6* alterations might have a slight effect on dentin or the abnormal enamel formation consequently affects dentin. The limitation of this study is a limited number of patient and tooth sample. More diseased tooth samples would clarify precise characteristics of the *ITGB6* teeth

## Conclusion

The study reports two novel *ITGB6* variants in a patient with AI and open bite, and, for the first time, hypertaurodontism and defective physical/mechanical properties of teeth in hypoplastic-hypomineralized AI. We expand the understanding of tooth defects and the genotypic and phenotypic spectra of autosomal recessive AI. The irregularity, weakness, and defective ultrastructure could cause *ITGB6* teeth prone to severe deterioration. We suggest that detailed phenotyping of affected individuals is important to delineate the phenotype of AI.

## Supplementary information

Supplementary Information
