# Supplementary material for: Novel ITGB6 variants cause hypoplastic-hypomineralized amelogenesis imperfecta and taurodontism: characterization of tooth phenotype and review of literature
Source: BDJ Open. 2023 Apr 11;9:15. doi: 10.1038/s41405-023-00142-y (PMC10090198; doi:10.1038/s41405-023-00142-y)
Supplement: Supplementary file 1 — Supplementary Information [file 41405_2023_142_MOESM1_ESM.pdf]

Supplementary Data

Table S1. A list of isolated and syndromic amelogenesis imperfecta-related genes according to the Human Phenotype Ontology (HP:0000705) used for genetic analysis in the study.

GENES	DISEASE_IDS
ACP4	OMIM:617297
AMBN	OMIM:616270
AMELX	OMIM:301200
AMTN	OMIM:617607
CLDN19	OMIM:248190,ORPHA:2196
CNNM4	OMIM:217080,ORPHA:1873
DLX3	OMIM:104510,OMIM:190320,ORPHA:3352
DNAJC21	ORPHA:811,OMIM:617052,OMIM:260400
ENAM	OMIM:204650,OMIM:104500
FAM20A	OMIM:204690,ORPHA:1031
FAM83H	OMIM:130900
GPR68	OMIM:617217
ITGB6	ORPHA:2850,OMIM:616221
KLK4	OMIM:204700
LAMA3	ORPHA:79404,ORPHA:79402,OMIM:226700,OMIM:619783,OMIM:619784, OMIM:245660
LAMB3	ORPHA:79404,ORPHA:79402,OMIM:226650,OMIM:226700,OMIM:104530
LTBP3	OMIM:617809,ORPHA:969,OMIM:601216
MMP20	OMIM:612529
ODAPH	OMIM:614832
ORAI1	OMIM:615883,OMIM:612782,ORPHA:3204,ORPHA:2593
PEX1	ORPHA:912,ORPHA:772,ORPHA:3220,ORPHA:44,OMIM:601539, OMIM:234580,OMIM:214100
PEX6	ORPHA:912,OMIM:614862,OMIM:616617,ORPHA:772,ORPHA:3220,

	ORPHA:95433,ORPHA:44,OMIM:614863
RELT	OMIM:618386
ROGDI	ORPHA:1946,OMIM:226750
SATB1	OMIM:619228,OMIM:619229
SLC10A7	OMIM:618363
SLC13A5	ORPHA:1946,OMIM:615905,ORPHA:442835
SLC24A4	OMIM:615887
SMARCD2	OMIM:617475
SP6	OMIM:620104
STIM1	OMIM:185070,OMIM:612783,ORPHA:3204,OMIM:160565,ORPHA:2593
TMEM165	OMIM:614727
WDR72	OMIM:613211

OMIM: Online Mendelian Inheritance in Man, ORPHA: Orphanet rare disease nomenclature.

Table S2. *In silico* analysis of the c.1661-3C>G variant in *ITGB6* using Human Splicing Finder (HSF).

Predicted signal	Predicted algorithm	cDNA Position	Interpretation
Broken Wildtype Acceptor Site	HSF Matrices[1]	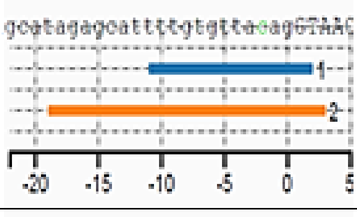	Alteration of the wildtype acceptor site, most probably affecting splicing.
	Maximum Entropy (MaxEnt)[1, 2]		

[1]Desmet FO, Hamroun D, Lalande M, Collod-Beroud G, Claustres M, Beroud C. Human Splicing Finder: an online bioinformatics tool to predict splicing signals. Nucleic Acids Res. 2009;37:e67–e67.

[2]Yeo G, Burge CB. Maximum entropy modeling of short sequence motifs with applications to RNA splicing signals. J Comput Biol. 2004;11:377–394.
